# Aldose reductase and glutathione in senile cataract nucleus of diabetics and non-diabetics

**DOI:** 10.1007/s10792-023-02776-1

**Published:** 2023-07-03

**Authors:** Kanishk Khare, Teena Mendonca, Gladys Rodrigues, Manjunath Kamath, Anupama Hegde, Shyamala Nayak, Ajay Kamath, Sumana Kamath

**Affiliations:** 1 Department of Ophthalmology, Kasturba Medical College, Mangalore, Manipal Academy of Higher Education (MAHE), Manipal, India; 2 Department of Biochemistry, Kasturba Medical College, Mangalore, Manipal Academy of Higher Education (MAHE), Manipal, India

**Keywords:** Senile cataract, Diabetes, Aldose reductase, Glutathione, HbA1c

## Abstract

**Purpose:**

The aim is to evaluate the aldose reductase (AR) and glutathione (GSH) activity in the nucleus of senile cataract in type 2 diabetes and non-diabetic group of patients.

**Methods:**

A total of 62 patients including 31 diabetics and 31 non- diabetics who were undergoing cataract surgery were included. Nucleus extracted was sent for analysis of AR and GSH activity while blood sample was taken for glycated haemoglobin (HbA1c) levels.

**Statistical analysis:**

Data were analysed using IBM SPSS 25. Comparison was carried out by unpaired *T*-test and correlations were established by Pearson’s correlation. The *p* value less than 0.05 was considered significant for all analyses.

**Study design:**

This is a prospective cross-sectional comparative study.

**Results:**

In this study, diabetic group patients showed earlier progression of cataract as compared to the non-diabetic group (*p*-value 0.0310). Mean HbA1c in the diabetic group was 7.34% compared to the non-diabetic group of 5.7% (*p* value < 0.001). AR in the diabetic patients was 2.07 mU/mg while the non-diabetic group was 0.22 mU/mg (*p*-value < 0.001). GSH in the diabetic group was 3.38 μMol/g and the non-diabetic group was 7.47 μMol/g (*p* value < 0.001). HbA1c showed positive correlation with AR among the diabetic group (*p*-value 0.028).

**Conclusion:**

Elevated oxidative stress can be strongly attributed to high AR and low GSH activity among the diabetic group as compared to the non-diabetic group and can lead to early cataract formation.

## Introduction

Cataract is the foremost cause of preventable visual impairment globally, contributing approximately 94 million cases [1]. Considering the socio-economic parameters, the prevalence among the low- and middle-income groups and regions contributes four times higher number of cases in the visual impairment than the higher-income groups and region [1]. With the global increase in lifespan and ageing population, cataract contributes to approximately 15 million cases above the age of 50 years causing blindness. Among the moderate to severe visual impairment (MSVI) group, cataract contributes to 78.8 million cases [1].

Aetiopathogenesis of acquired cataract is multifactorial. Numerous studies have contributed towards the genetic, nutritional, biochemical, epidemiological and photochemical aspects of development of cataract. Various studies have been carried out categorize the etiological factors on the basis of different morphological development of cataract, namely cortical, nuclear sclerosis and subcapsular type [2].

Major pathogenesis contributing to senile cataract includes breakdown and protein aggregation, damage to fibre cell membranes, glutathione deficiency, damage by oxidative stress, calcium levels elevation and abnormal migration of lens epithelial cell [3]. The risk factors that may trigger or accelerate such events includes UV radiations, smoking, diabetes, dehydration crisis, oxidative stress, hyperlipidaemia, etc. [4] to name a few.

One of the key biochemical factor maintaining the oxidative balance in the lens is the reducing agent glutathione (GSH). In various studies it has found that GSH is one of the most vital anti-oxidant factor of the lens [5]. The synthesis and recycling of glutathione falls with age [6].

Patients with diabetes mellitus have been found at risk of early cataract development up to five times as compared to non-diabetics [6]. Various studies have been carried out in the past highlighting the role of sorbitol or polyol pathway in cataract formation and progression among the diabetic patients [7]. In the patients with poor glycaemic control, polyol pathway is activated to process the glucose. Aldose reductase (AR) is the crucial enzyme in this process. Sorbitol and fructose accumulation in the lens epithelium and lens fibres is reflected as osmotic trauma, leading to lens opacification [7].

Previous studies have measured the value of glutathione and aldose reductase in the blood of patients and correlated the levels in the diabetic population and non- diabetic population [8]. Few studies on animal models have been conducted to determine the levels of these enzymes in the nucleus also [9]. This study emphasizes the oxidative stress in the nucleus of senile cataract among the two group of patients by measuring and comparing these two biochemical parameters in the lens and correlating their values with the glycaemic control.

## Materials and methods

### Study setting

Kasturba Medical College, Government Wenlock Hospital, Mangalore and Department of Biochemistry.

### Study design

Prospective cross-sectional comparative study.

### Study participants

Patients undergoing small incision cataract surgery.

### Inclusion criteria for cases

All senile cataract patients undergoing cataract surgery who are a known case of type-2 diabetes and on medication.

### Inclusion criteria for controls

All senile cataract patients undergoing cataract surgery who are not a known case of type-2 diabetes.

### Exclusion criteria for cases and controls:

1. Cataract in age less than 50 years.
2. Type-1 diabetics.
3. Complicated, traumatic and congenital cataracts.

### Study period

October, 2019–September, 2021.

### Sample size

With 95% confidence level and 90% power with respect to reference article, sample size is 62 cases: 31 patients in diabetic group and 31 patients in the non- diabetic group with cataract.

$$N = 2\;(Z_{\alpha } \times Z_{\beta } )2 \times \delta_{2} /d_{2}$$

*Zα* = 1.96 at 95% confidence level.

*Zβ* = 1.28 at 90% power.*δ* = 792.76, *d* = 650 (w.r.t. Reference article [8])

### Sampling method

Non-random convenience sampling.

### Data analysis

It was carried out by descriptive statistics. All the data were tabulated in excel sheet, followed by analyzing using IBM SPSS 25. Descriptive and categorical data have been represented as means ± std deviation and percentages, respectively. The *T*-test was used to compare mean age, RBS, HbA1c, AR, and GSH among the diabetic group of patients and non-diabetic group of patients, whereas, the variables gender, and cataract types between the two groups were compared using Chi-square analysis. Additionally, within the diabetic group, the mean HbA1c, AR, and GSH values across the diabetic retinopathy groups were also compared using *T*-test. The correlations were evaluated by Pearson’s correlation between the age, duration of diabetes, and HbA1c each with AR and GSH levels. Statistical significance was based on the *p* value, with less than 0.05 considered significant for all analyses.

### Data collection tool

- This study was conducted on patients with the senile cataract undergoing cataract surgery.
- An informed consent was taken.
- Detailed history, including the duration of diabetes and medication history, was taken followed by ophthalmic examination including visual assessment, anterior segment evaluation, dilated fundus examination using indirect ophthalmoscope.
- Nucleus extracted during cataract surgery will be divided into two parts and transferred into two buffers, namely Na + K + biphosphate buffer and normal saline, to estimate the activity of aldose reductase and glutathione, respectively.
- The nucleus was homogenized in a 1 ml volume of the same buffer. Homogenates were centrifuged at 27,000 × g for 20 min at 4 °C, and the supernatant was assayed for AR. AR was assayed by the method of Hayman et al. [10]
- To determine lens GSH, 0.2 mL of the homogenized lens sample was added directly into tubes containing 0.2 mL of 1 mol/L perchloric acid supplemented with 2 mmol/L of disodium EDTA, vortex-mixed, and centrifuged for 5 min at 10,000 g (4 °C) to remove the protein precipitate. Lens GSH was determined in a clear supernatant using Ellman’s reagent [7]. 0.2 mL of this supernatant, 0.8 mL of 0.3 mmol/L Na2HPO4, and 0.2 mL of 5,5*_* -dithiobis-2nitrobenzoic acid in 1% sodium citrate were added in succession. The intensity of the resulting yellow colour was read spectrophotometrically at 410 nm. [11]
- Blood sample was collected for HbA1c, and values were noted.

The study was approved by the Institutional Ethics Committee of Kasturba Medical College, Mangalore. (IEC KMC MLR 10-19/473).

## Results

### Patient demographics

In our study total 62 patients were included, comprising 31 diabetics and 31 non-diabetics. The diabetic group patients mean age was 60.29 years (standard deviation of 6.59 years), while non-diabetic group was 64.87 years (standard deviation of 9.45 years) with *p*-value of 0.031, which is clinically significant.

The gender distribution in both the groups showed marginally a greater number of females. Diabetic group included 16 females and15 males, while the non- diabetic group comprised of 17 females and 14 males (*p* value 0.79).

### Clinical and blood parameters

The distribution of type of cataract in diabetic group depicted maximum number of cases showing mixed type (45.2%), that is, with both nuclear sclerosis and posterior subcapsular opacification. Non-diabetic group had comparable number cases in each type of morphological distribution. The mean DM duration was 4.3 ± 3.06 years among 31 diabetics (*p*-value 1). On treatment front, 28(90.3%) among them were on orally administered anti-hyperglycaemic medication while only 3(9.7%) were on insulin formulation (Table 1).

**Table 1:** Comparison of distribution of cataract types using chi-square analysis

Group	*n*	Nuclear sclerosis	Posterior subcapsular + nuclear sclerosis	Senile mature cataract	Chi square (*p* value)
Diabetic	Count	6	14	11	1.66 (0.43)
	%	19.4%	45.2%	35.5%	
Non- diabetic	Count	10	10	11	
	%	32.3%	32.3%	35.5%	

The mean HbA1c value in the diabetic group was 7.34% compared to the non- diabetic group of 5.7% with *p* value < 0.001 which was extremely significant (Table 2).

**Table 2:** Mean HbA1c and RBS value among the diabetics and non-diabetics

Variable	Group	*N*	Mean	Std. deviation	*T* statistic	*p* value
HbA1c (%)	Diabetic	31	7.339	1.3856	5.767	< 0.001
	Non-diabetic	31	5.700	0.7638		
RBS (mg/dL)	Diabetic	31	151.26	43.347	5.441	< 0.001
	Non-diabetic	31	101.58	26.564		

The mean random blood sugar (RBS) value in the diabetic group was 151 mg/dl compared to the non-diabetic group of 101 mg/dl with *p*-value < 0.001 which was extremely significant.

### Biochemical parameters

Mean aldose reductase (AR) in the diabetic patients was 2.07 mU/mg of protein while the non-diabetic group was 0.22 mU/mg of protein with *p*-value less than 0.001. Mean glutathione activity in the diabetic group was 3.38 μMol/g of lens and the non-diabetic group was 7.47 μMol/g of lens (*p* value < 0.001) (Table 3).

**Table 3:** Mean AR and GSH activity

Variable	Group	*N*	Mean	Std. deviation	*T* statistic	*p* value
AR (mU/mg protein)	Diabetic	31	2.0713	1.53634	6.693	0.001
	Non-diabetic	31	0.2161	0.14495		
GSH (μMol/g lens)	Diabetic	31	3.3761	1.02500	− 16.377	0.001
	Non-diabetic	31	7.4748	0.94399		

### Correlations

**Fig. 1:**
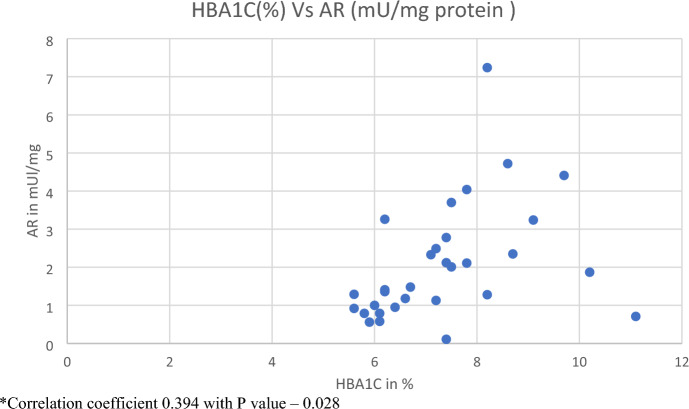
Correlation between HbA1c and AR activity within the diabetic patients. *Correlation coefficient 0.394 with *p* value – 0.028

HbA1c values were correlated with the AR and GSH activity. HbA1c had a positive correlation with AR among the diabetic group with *p*-value of 0.028 (Table 4 and Fig. 1).

**Table 4:** Correlation of HBA1C with AR and GSH levels using Pearson’s correlation

Variables compared	Correlation	Diabetics	Non-diabetics
HbA1c versus AR	Correlation coefficient	0.394	− 0.126
	*p* value	0.028	0.501
HbA1c versus GSH	Correlation coefficient	− 0.040	0.127
	*p* value	0.831	0.497

**Fig. 2:**
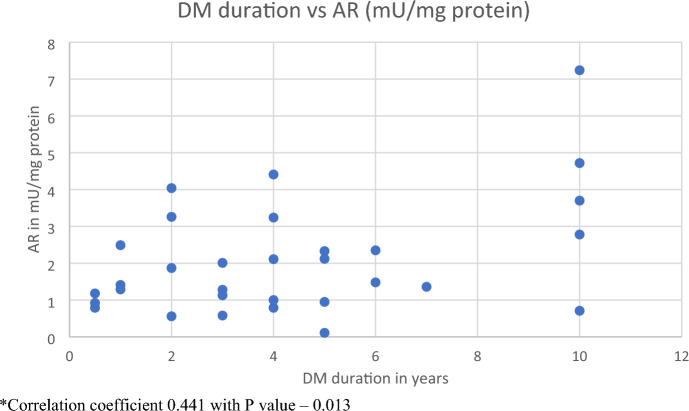
Correlation between DM duration and AR within the diabetic patients. *Correlation coefficient 0.441 with *p* value – 0.013

Duration of type 2 diabetes mellites was correlated with the AR activity. Correlation coefficient was 0.44 which was significant with *p* value of 0.013. However, GSH activity had no significant correlation (Table 5 and Fig. 2).

**Table 5:** Correlation of duration of diabetes with AR and GSH levels in the diabetic group using Pearson’s correlation

Variables compared	Correlation	Diabetics
DM duration versus AR	Correlation coefficient	0.441
	*p* value	0.013
DM duration versus GSH	Correlation coefficient	0.154
	*p* value	0.408

## Discussion

Various studies performed in the past have indicated rapid and frequent cataract progression in the presence of diabetes. Framingham Eye study reported higher prevalence of cataract up to three times in the diabetic patients aged below 65 years as compared to their non-diabetic counterparts [12]. Among people above 65 years of age cataract was more frequent among diabetics than non-diabetics [12]. Klein et al. [13] in their study found that diabetic patients showed earlier development of cataract than non-diabetic patients.

In the present study, the mean age of patients with cataracts was less in the diabetic group than the non-diabetic group and was statistically significant. Both the groups had marginally a greater number of female patients, 51.6% and 54.8%, respectively, as compared to the males in both the groups, 48.4% and 45.2%, respectively, though the values were not statistically significant (Table 2). Nevertheless, this is in accordance with the epidemiological data that states cataract is more widespread in women than men [14].

Morphologically, diabetes has been associated with cortical and subcapsular opacification of lens. In follow-up of a large cohort of the Beaver Dam Eye Study, they elicited the relationship between diabetes and cataract morphology. It showed an association between diabetes and incidence of posterior subcapsular cataract and cortical type of cataract [6]. In the present study, among the diabetic group maximum number (45.2%) of patients had mixed morphology of opacification including posterior subcapsular and nuclear sclerosis (Table 1). While the non-diabetic group showed comparable distribution of cataract on the basis of morphology (Table 1), values were not statistically significant. Nevertheless, this study showed similar trends as seen in the Blue Mountain Eye Study which reported increased incidence of posterior subcapsular type in diabetics [15].

The primary outcome of the present study is aldose reductase and glutathione activity in the crystalline lens nucleus. Polyol or sorbitol pathway has been extensively studied in the past for its role in cataract formation among the diabetic patients. However, this is the first study carried out to evaluate the aldose reductase (AR) and Glutathione (GSH) activity in the nucleus of senile cataract in type 2 diabetes and non-diabetic group of patients.

The first enzyme involved in this pathway is aldose reductase. It is the key initiator of sorbitol formation in the lens [16]. Sorbitol accumulation further increases the oxidative stress and causes opacification of lens fibres. Various animal models have been studied that have demonstrated the role of aldose reductase in cataract formation after inducing diabetes by either pancreatomy or chemical ablation of the beta cells responsible of insulin production in the pancreas [9]. Sorbitol accumulation also causes osmotic stress to the main centre of protein synthesis, endoplasmic reticulum (ER) [16, 17]. With fluctuation of glucose in the diabetics, ER also initiates unfolded protein response which causes reactive oxygen species (ROS) generation [7, 17]. These free radical scavengers will not only lead to damage to lens fibres, but also causes more consumption and henceforth reduction in GSH levels.

Bhatia et al. conducted a study among the senile cataract patients comparing the levels of aldose reductase in the blood of diabetics, non-diabetics and control group [8]. They found highest mean levels of AR in the diabetic group and was statistically significant [8]. In this study, the mean AR activity of lens nucleus among the diabetics was 2.07 mU/mg of protein as compared to 0.21 mU/mg of protein in the non-diabetic group. This was highly statistically significant with the *p* value of < 0.001 (Table 3). Apart from the AR activity, studies conducted on aldose reductase inhibitors (ARI) have also show regression of cataract in presence of diabetes in the animal models [18]. Hence, this again ascertains the role of AR in the cataract formation among the diabetic patients.

The cataract progression risk has been positively correlated in numerous studies with the duration of diabetes and poor glycaemic control among the diabetics [8, 19]. In the present study, the mean duration of diabetes was 4.3 years. Duration of diabetes had a significant positive correlation with the aldose reductase activity with value of 0.44 and *p* value of 0.03. This hence helps in eliciting the role of aldose reductase and the significance of duration of diabetes among the diabetic patients.

Studies have been carried out investigating the level of aldose reductase and glutathione in blood of the patients [8, 19]. Aldose reductase has been more frequently explored in the blood of diabetic patients [20]. Glutathione, on the other hand has been a key anti-oxidant and its value is of more significance in the ageing or senile cohort [4]. The mean GSH activity in the current study was analysed and compared among the diabetics and non-diabetics. Diabetic patients showed mean GSH activity of 3.38 μMol/g of lens which was far less than the non-diabetic group with mean of 7.48 μMol/g of lens. (Table 2). These data were substantially significant statistically with *p* value of < 0.001. Hence, lower levels of anti-oxidants further aggravate the oxidative stress in the lens.

The American Diabetes Association recommended glycated haemoglobin (HbA1c) as the measure of glycaemic control for the last 6–8 weeks of a patient [21]. This can also serve as an alternative to diagnosis made by the fasting blood glucose levels. HbA1c of 6.5% has been recommended as a cut-off value to diagnose diabetes [22]. In this study, the glycated haemoglobin was taken as a marker of glycaemic control in the two groups. The diabetic group showed an elevated mean HbA1c levels of 7.34% as compared to the non-diabetic group with mean HbA1c value of 5.7% (Table 2). These results were consistent with the results previous large cohort of the Beaver Dam Eye Study which stated higher HbA1c levels were strongly associated with significant risk of cortical and nuclear cataracts [6]. Similar results have been reflected in this study also. We also recorded the values of random blood sugar in each patient. The mean random blood sugar (RBS) value in the diabetic group was 151 mg/dl compared to the non-diabetic group of 101 mg/dl with *p* value < 0.001 which was highly significant (Table 2). However, in this study we have strictly adhered to the values of HbA1c as a marker of glycaemic control and for further statistical correlations among variables. The values of AR and GSH found in this study were further correlated with the HbA1c levels among the diabetics and non-diabetics. AR activity showed a noteworthy positive correlation (correlation coefficient 0.394) with glycated haemoglobin in the diabetic group. This was significant with *p* value of 0.028 (Table 4). On the other hand, non-diabetic group showed a negative correlation between AR and HbA1c, though it was not significant (Table 4). GSH activity showed no significant correlation with the HbA1c in the present study. Similar results have been reflected in a study by Bhatia et al. who correlated serum levels of AR, GSH and HbA1c in senile cataract patients [8].

This study hence demonstrates that the diabetic group showed poor glycemic control and had significantly increased aldose reductase activity and low glutathione levels which may have contributed to earlier cataract formation as compared to their non-diabetic counterpart.

## Conclusion

The present study helps to establish a contribution of the Aldose reductase and glutathione activity in the lens nucleus and formation of cataract. Variables compared between the diabetic and non-diabetic groups were found to have significant value. Poor glycaemic control seen in diabetes triggers increased AR activity. Increased level of AR among the diabetic group extensively contributed to the oxidative stress in the lens. This was further worsened by the low levels of protective antioxidant glutathione whose activity was substantially less in the diabetic group as on comparison to the non-diabetic group. Elevated oxidative stress can hence be attributed to high AR and low GSH activity and can lead to early cataract formation.
